# ROS/Enzyme Dual-Responsive Drug Delivery System for Targeted Colorectal Cancer Therapy: Synergistic Chemotherapy, Anti-Inflammatory, and Gut Microbiota Modulation

**DOI:** 10.3390/pharmaceutics17070940

**Published:** 2025-07-21

**Authors:** Xin Zhang, Ruonan Lian, Bingbing Fan, Lei Meng, Pengxia Zhang, Yu Zhang, Weitong Sun

**Affiliations:** 1 College of Pharmacy, Jiamusi University, Xiangyang District, Jiamusi 154007, China; zx2644688602@163.com (X.Z.); lianrn@163.com (R.L.); fan2159129178@163.com (B.F.); menglei13149663633@163.com (L.M.); pengxiaz@jmsu.edu.cn (P.Z.); zhangyu@jmsu.edu.cn (Y.Z.); 2 Heilongjiang Center for Drug Evaluation and Inspection, Harbin 150000, China

**Keywords:** colorectal cancer, synergistic treatment, prodrug micelles, microsphere, ROS/enzyme dual-responsive, inflammations, gut microbiota

## Abstract

**Objectives:** Colorectal cancer (CRC) is a leading cause of cancer-related mortality, driven by chronic inflammation, gut microbiota dysbiosis, and complex tumor microenvironment interactions. Current therapies are limited by systemic toxicity and poor tumor accumulation. This study aimed to develop a ROS/enzyme dual-responsive oral drug delivery system, KGM-CUR/PSM microspheres, to achieve precise drug release in CRC and enhance tumor-specific drug accumulation, which leverages high ROS levels in CRC and the β-mannanase overexpression in colorectal tissues. **Methods:** In this study, we synthesized a ROS-responsive prodrug polymer (PSM) by conjugating polyethylene glycol monomethyl ether (mPEG) and mesalazine (MSL) via a thioether bond. CUR was then encapsulated into PSM using thin-film hydration to form tumor microenvironment-responsive micelles (CUR/PSM). Subsequently, konjac glucomannan (KGM) was employed to fabricate KGM-CUR/PSM microspheres, enabling targeted delivery for colorectal cancer therapy. The ROS/enzyme dual-response properties were confirmed through in vitro drug release studies. Cytotoxicity, cellular uptake, and cell migration were assessed in SW480 cells. In vivo efficacy was evaluated in AOM/DSS-induced CRC mice, monitoring tumor growth, inflammatory markers (TNF-α, IL-1β, IL-6, MPO), and gut microbiota composition. **Results:** In vitro drug release studies demonstrated that KGM-CUR/PSM microspheres exhibited ROS/enzyme-responsive release profiles. CUR/PSM micelles demonstrated significant anti-CRC efficacy in cytotoxicity assays, cellular uptake studies, and cell migration assays. In AOM/DSS-induced CRC mice, KGM-CUR/PSM microspheres significantly improved survival and inhibited CRC tumor growth, and effectively reduced the expression of inflammatory cytokines (TNF-α, IL-1β, IL-6) and myeloperoxidase (MPO). Histopathological and microbiological analyses revealed near-normal colon architecture and microbial diversity in the KGM-CUR/PSM group, confirming the system’s ability to disrupt the “inflammation-microbiota-tumor” axis. **Conclusions:** The KGM-CUR/PSM microspheres demonstrated a synergistic enhancement of anti-tumor efficacy by inducing apoptosis, alleviating inflammation, and modulating the intestinal microbiota, which offers a promising stimuli-responsive drug delivery system for future clinical treatment of CRC.

## 1. Introduction

Colorectal cancer (CRC) is a globally prevalent and highly malignant inflammation-related tumor with high morbidity and mortality [1,2]. The CRC microenvironment is complex, where colorectal cancer cells are affected by multiple factors such as intestinal microbiota dysbiosis, chronic inflammation, and tumor microenvironment (e.g., elevated reactive oxygen species, hypoxia, and low pH) [3,4]. At present, the main treatment options for CRC include surgery, radiotherapy, and chemotherapy. Among these, chemotherapy is the most widely used due to its ability to achieve targeted and combination therapy, effectively controlling the development of CRC [5,6]. Conventional chemical drug chemotherapy has poor selectivity, which can lead to intestinal microbiota imbalance and serious side effects [7,8]. Therefore, it is of great significance to find a reasonable and effective drug treatment regimen for CRC.

Curcumin (CUR), a lipophilic polyphenol derived from *Curcuma longa*, has multiple pharmacological effects such as anti-inflammatory, antioxidant, and anti-tumor effects [9,10]. As a potent anti-colorectal cancer (CRC) agent, CUR targets multiple signaling pathways: it suppresses STAT3 and NF-κB activity, thereby downregulating downstream effectors (e.g., COX-2, cyclin D1, Bcl-2, and Bcl-XL). Moreover, CUR modulates key oncogenic drivers by reducing EGFR/HER-2 expression, inhibiting IGF-1R-mediated AKT phosphorylation, and blocking the Wnt/β-catenin pathway via downregulation of β-catenin, TCF4, and vimentin. These multifaceted actions collectively induce cell cycle arrest, suppress proliferation, and promote apoptosis [11,12,13]. In addition, CUR can eliminate active oxygen free radicals, increase the activity of catalase and glutathione peroxidase, exert antioxidant effects, and have a significant inhibitory effect on peroxidative damage [14]. However, despite its therapeutic potential, CUR’s clinical application is limited by its poor water solubility and low bioavailability, which compromise its efficacy as a monotherapy. Notably, studies have shown that CUR synergizes with conventional chemotherapy drugs, offering a promising combinatorial strategy for cancer treatment [15,16,17].

Mesalazine (MSL), a first-line anti-inflammatory drug, has been reported to effectively inhibit cyclooxygenase (COX) activity, which affects prostaglandin production and reduces the inflammatory response in the colon [18,19]. Inflammation is a crucial component of the tumor microenvironment; thus, MSL can improve the tumor microenvironment by inhibiting inflammatory responses, making it easier for CUR to exert its anti-tumor effect [20]. Furthermore, it has been shown that MSL can also suppress the growth of CRC tumor cells by decreasing the expression of Cyclin D1, activating the PPAR-g and AMPK pathways, and inhibiting the wntβ-catenin pathway [21,22]. Therefore, in order to better exert the anti-tumor efficacy of CUR, CUR and MSL were synergistically administered in this study with the aim of synergistically improving the anti-tumor effect by promoting apoptosis, anti-inflammation, and regulating the intestinal microbiota.

The prodrug system couples the drug with the polymer carrier through chemical bonds to form polymer compounds [23]. Commonly used carriers are prodrug liposomes, micelles, gels, etc. [24]. Compared to other carriers, polymer micelles have many attractive advantages, which can dramatically improve the solubility, stability, and bioavailability of hydrophobic anticancer drugs [25,26,27]. In addition, the drug in the polymer prodrug micelle system can participate in the construction of the polymer prodrug micelle as a part of the carrier, realizing the dual function identity of the drug as the active ingredient and the carrier [28,29]. However, traditional passive targeting has not achieved the desired anti-tumor effect, so it is of great significance to develop a more “intelligent” nano-drug delivery system to achieve efficient treatment of tumors [30,31]. After the stimuli-responsive nano-drug delivery system reaches the tumor site, it responds to certain specific stimuli (such as GSH, pH, enzymes, ROS, etc.) to accurately release drugs and improve the targeting of the nanosystem [32,33,34,35].

Accordingly, this study aimed to address the bottleneck problem of chemical drug treatment in CRC to construct a stimuli-responsive prodrug micellar system. Central to this system is the design of a novel reactive oxygen species (ROS)-responsive amphiphilic polymer prodrug, polyethylene glycol monomethyl ether-thioether-MSL (PSM). In PSM, polyethylene glycol monomethyl ether (mPEG) serves as the biocompatible hydrophilic segment, while the non-steroidal anti-inflammatory drug mesalazine (MSL) acts as both the therapeutic agent and the hydrophobic segment, achieving a dual carrier-drug functionality. Crucially, MSL is conjugated to mPEG via a ROS-sensitive thioether linker, enabling specific disassembly and drug release triggered by the elevated ROS levels characteristic of the CRC tumor microenvironment. Building upon this PSM prodrug polymer, the stimuli-responsive prodrug system curcumin/mPEG-S-MSL (CUR/PSM) micelles were successfully constructed by encapsulating curcumin (CUR) into the hydrophobic core of PSM micelles via the thin-film hydration method. This design integrates the anti-tumor effects of CUR with the anti-inflammatory properties of MSL within a single, tumor-targeted nanocarrier.

For the treatment of CRC, oral administration is a common approach, which can make the drug directly act on the colorectal tissue to maximize the anti-tumor effect. However, oral drugs often encounter problems such as pH changes, digestive enzymes, and physical extrusion before reaching the target site [36]. Therefore, the most ideal drug delivery option is to design a carrier that can precisely deliver drugs to the intestinal tumor site in response to the complex intestinal environment to achieve targeted therapy. The colorectum contains a rich microbial community and abundant specific enzymes, which can specifically degrade some natural polysaccharides, so using materials with enzymatic degradation properties is one of the simplest and most effective strategies. At present, konjac glucomannan (KGM) is a colon-specific drug delivery carrier, which significantly influences the advancement of carriers for colon-specific drug delivery [37]. It underwent specific degradation solely by β-mannanase present in the colon, and has excellent properties such as promoting intestinal physiological peristalsis, alleviating intestinal inflammation, regulating intestinal microbiota, and treating tumors [38].

**Figure 1:**
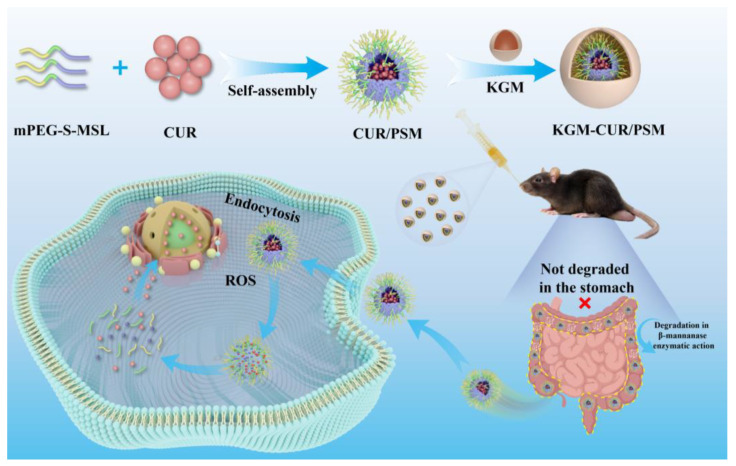
Schematic of KGM-CUR/PSM microsphere preparation and its dual-responsive therapeutic action in colorectal cancer. The preparation involves synthesizing ROS-responsive prodrug polymer mPEG-S-MSL (PSM) and loading it with curcumin (CUR) to form CUR/PSM micelles, which are encapsulated in konjac glucomannan (KGM) microspheres. Upon oral administration, microspheres protect the payload in the upper GI tract, with KGM degrading via colonic β-mannanase to release CUR/PSM micelles at the tumor site. Elevated tumor ROS cleaves the thioether bond in PSM, triggering release of active CUR and mesalazine (MSL). The released CUR exerts anti-CRC effects by inducing apoptosis, inhibiting proliferation, and modulating gut microbiota, while MSL provides anti-inflammatory action. KGM metabolites further modulate microbiota, synergistically disrupting the “inflammation-microbiota-tumor” axis.

Based on the above advantages, CUR/PSM micelles were encapsulated into KGM-CUR/PSM microspheres by KGM. Consequently, the enzymatic response in the colorectal environment and the ROS response at the tumor site were combined to achieve a dual-response effect, resulting in dual-targeting roles for colorectal and cancer localization. At the same time, the mechanism of action of CUR, MSL, and KGM in the synergistic treatment of CRC was explored, such as regulating the intestinal microbiota, inhibiting the inflammatory environment, and improving the anti-tumor effect, which provided a feasible research idea and treatment method for the drug treatment of CRC. The preparation process and therapeutic mechanism of KGM-CUR/PSM are illustrated in Figure 1.

## 2. Materials and Methods

### 2.1. Materials and Reagents

Polyethylene glycol monomethyl ether (mPEG), 4-Dimethylaminopyridine (DMAP), pyrene, and N,N′-dicyclohexylcarbodiimide (DCC) were purchased from Macklin Biochemical Co., Ltd. (Shanghai, China). Mesalazine (MSL), curcumin (CUR), N-hydroxybutanediimide (NHS), 2,2′-Thiodiacetic acid (TDA) were obtained from Aladdin Co., Ltd. (Shanghai, China). Konjac Glucomannan (KGM) was bought from Bomei Biological Co., Ltd. (Hefei, China). Fetal bovine serum (FBS), phosphate-buffered saline (PBS), penicillin-streptomycin solution, cell lysate (RIPA), Cy5-NH_2_ and 4,6-diamidino-2-phenylindole (DAPI), trypsin, antibodies, and the CCK-8 cell kit were acquired from Biyuntian Biotechnology Co., Ltd. (Shanghai, China). Sheep anti-rabbit horseradish peroxidase-labeled secondary antibody was purchased from Invitrogen Co., Ltd. (Carlsbad, CA, USA). Azoxymethane (AOM) was obtained from Sigma Co., Ltd. (St. Louis, MO, USA). Dextran sodium sulfate (DSS) was bought from Meilun Biotechnology Co., Ltd. (Dalian, China). RPMI 1640 medium was obtained from Gibco Co., Ltd. (Grand Island, NY, USA). All other reagents were of analytical or chromatographic grade. The key compounds used in this study and their characteristics are summarized in Table 1

### 2.2. Cells and Animals

The human colorectal cancer cell line SW480 was obtained from the College of Preventive Medicine at Jiamusi University (Jiamusi, China). Cells were cultured in RPMI 1640 medium (Gibco, Carlsbad, CA, USA) supplemented with 10% FBS (Biyuntian Biotechnology, Shanghai, China) and 1% penicillin-streptomycin (Biyuntian Biotechnology, Shanghai, China). The cells were maintained at 37 °C in a humidified atmosphere containing 5% CO_2_. Male C57BL/6 mice, aged 4 to 6 weeks and weighing between 20 and 22 g, were obtained from Changsheng Biotechnology Company in Liaoning, China. The animal experiments and feeding were conducted under specific pathogen-free (SPF) conditions, adhering to protocols approved by the Animal Protection and Use Committee of Jiamusi University (Ethics No. JMSU-2023113001).

### 2.3. Synthesis and Characterization of PSM and PM

#### 2.3.1. Synthesis of 2,2′-Thiodiacetic Acid Anhydride (TDAA)

Notably, 4 g of 2,2′-thiodiacetic acid (TDA) was dissolved in 25 mL of acetyl chloride. The solution was heated to 51 °C and refluxed for 2 h. Then, acetyl chloride was removed under reduced pressure at 45 °C. The reaction product was precipitated and purified with cold ether. After filtration and drying in a vacuum furnace, the product TDAA was obtained (the yield of TDAA was 82%).

#### 2.3.2. Synthesis of Polyethylene Glycol Monomethyl Ether-Thioether-Carboxylic Acid (mPEG-S-COOH)

Notably, 2 g of mPEG (MWCO = 1900 Da), 0.132 g of TDAA, and 0.4 g of DMAP were dissolved in 20 mL of DMSO. After reacting for 48 h at room temperature, the resulting solution was dialyzed using a dialysis bag (MWCO = 3500 Da) for 48 h in DI water. The liquid in the dialysis bag was filtered to remove impurities. The product (1.85 g, yield 92.5%) was freeze-dried using a freeze dryer (Advantage EL-85, VirTis Ltd., Gardiner, NY, USA).

#### 2.3.3. Synthesis of mPEG-S-MSL (PSM)

Notably, 1 g of mPEG-S-COOH, 0.2 g of DCC, and 0.2 g of NHS were dissolved in 10 mL DMSO, stirred at room temperature for 2 h, followed by addition of 0.08 g of MSL, and further reacted for 72 h. Then, the resulting product (0.95 g, yield 89%) was obtained after dialysis and freeze-dried using a freeze dryer (Advantage EL-85, VirTis Ltd., USA).

#### 2.3.4. Synthesis of mPEG-MSL (PM)

PM micelles were synthesized using the following method. Briefly, 2 g of mPEG and 0.4 g of DMAP were dissolved in 20 mL of DMSO. The mixture was stirred at room temperature for 2 h and 0.08 g of MSL was then added. Finally, the resulting solution was reacted for 72 h. The product (1.71 g, yield 82%) was harvested by dialysis and freeze-dried by a freeze dryer (Advantage EL-85, VirTis Ltd., USA).

#### 2.3.5. Characterization of PSM and PM

The ^1^H NMR spectra of PSM and PM were determined using ^1^H NMR spectrometer (AV600, Bruker Ltd., Karlsruhe, Germany) at 25 °C. Samples were dissolved in chloroform with tetramethylsilane (TMS) as an internal reference.

The mass spectrometry analysis of mPEG-S-COOH, PSM, and PM was performed using a MALDI-TOF mass spectrometer (Autoflex, Bruker Ltd., Germany) equipped with a nitrogen laser (337 nm).

The FTIR spectra of PSM and PM were acquired using a Fourier-Transform Infrared spectrometer (Spectrum100, PerkinElmer Ltd., Beaconsfield, UK). Each sample was thoroughly mixed with 100 mg of potassium bromide (KBr), ground into a fine powder, compressed into pellets, and scanned within the wavenumber range of 4000–500 cm^−1^.

### 2.4. Determination of the Critical Aggregation Concentration (CAC)

The critical aggregation concentration (CAC) of PSM polymers was assessed by the pyrene fluorescent probe method. In brief, 1 mg mL^−1^ of pyreneacetone solution was introduced into a set of glass bottles. After drying with nitrogen, different concentrations of PSM were introduced into each bottle and then protected from light for 24 h. The excitation spectrum (200~500 nm) was measured at an excitation wavelength of 334 nm by fluorescence spectrophotometer (F95S, LingGuang Ltd., Shanghai, China), and the fluorescence intensity at 373 nm (I_1_) and 384 nm (I_3_) was recorded. With the ratio of I_1_/I_3_ as the ordinate and logC as the horizontal coordinate, the CAC value of PSM was analyzed and calculated [39].

### 2.5. Synergistic Effect of the Two Drugs at Different Ratios

The CCK-8 assay was employed to assess the inhibitory effects of CUR and MSL on the proliferation of SW480 cells. Cells in the logarithmic growth phase were seeded into 96-well plates for incubation. When the cell fusion rate reached 70–80%, the culture medium with drug solutions of different concentrations (CUR:MSL = 0.5:1, 1:1 1.5:1, 2:1, 2.5:1) was added. For the combination groups, the concentrations (10, 20, 30, 40, 50 μM) refer to the total concentration of CUR and MSL combined. For the single-drug groups (CUR alone and MSL alone), the concentrations (10, 20, 30, 40, 50 μM) refer to the concentration of the individual drug. After incubation for 48 h, 10 µL of CCK-8 solution was introduced into each well. The cells were further incubated for 2 h and the absorbance OD was determined at 450 nm by using an enzyme meter (Xsite, ENVISION Ltd., UK) under light-protected conditions and the cell inhibition rate was computed by the following formula: inhibition (%) = (OD_control_ − OD_sample_)/(OD_control_ − OD_blank_) × 100%. The 50% inhibitory concentration (IC_50_) values were calculated for each group using Graphpad Prism (Version 9.0), and the Combination Index (CI) was computed using CalcuSyn software (Version 2.0) based on the Chou-Talalay method to investigate the synergistic impact of the two drugs on SW480 cells (CI < 1, synergistic effect; CI = 1, additive effect; CI > 1, antagonistic effect).

### 2.6. Preparation and Characterization of CUR/PSM Micelles

CUR/PSM micelles in this study were prepared via the thin-film dispersion technique. Notably, 1 mg of CUR and 10 mg of the polymer PSM were dissolved in 10 mL of methanol. The mixture was sonicated until complete dissolution. The solvent was removed by rotary evaporation at 45 °C until a uniform thin film formed on the flask wall. Then, 10 mL of purified water was added for hydration and stirred for 40 min at a water bath temperature of 40 °C. Finally, the CUR/PSM micelles were obtained by membrane decontamination. CUR/PM micelles were formulated using an identical procedure.

The particle size, polydispersity index (PDI), and zeta potential were determined by dynamic light scattering (DLS, Nano-s, Malvern Instruments Ltd., Malvern, UK) at 25 °C. The CUR/PSM micelles were filtered and diluted with purified water before being loaded into dedicated cuvettes for size measurement and zeta potential cells for zeta potential analysis.

Transmission electron microscopy (TEM, HT7700, Hitachi Ltd., Tokyo, Japan) was used to characterize the morphology of CUR/PSM micelles. TEM samples were prepared by depositing a drop of micelle solution onto a carbon-coated copper grid, followed by negative staining with 2% (*w*/*v*) phosphotungstic acid and air-drying before observation at an accelerating voltage of 80 kV.

Additionally, differential scanning calorimetry analysis (DSC, STA409PC, Hengjiu Ltd., Xuzhou, China) was performed under a nitrogen atmosphere (flow rate: 50 mL/min) with a heating rate of 10 °C/min over a temperature range of 25–300 °C.

X-ray diffraction analysis (XRD, Geigerflex, Rigaku Ltd., Tokyo, Japan) was conducted using Cu-Kα radiation (λ = 1.5406 Å) at 40 kV and 30 mA, with a scanning rate of 4°/min and a 2θ range of 3–50° to determine that CUR was successfully encapsulated by PSM.

The drug loading (DL%) and encapsulation efficiency (EE%) of the CUR/PSM micelles were assayed at 425 nm via an HPLC system (Welch Materials column, 4.6 × 250 mm, 5 μm; mobile phase: methanol: 5% glacial acetic acid, 70:30 *v*/*v*; flow rate: 1.0 mL·min^−1^; column temperature: 25 °C; detection wavelength: 425 nm; injection volume: 10 μL). The DL% and EE% of CUR in the CUR/PSM micelles were obtained by using the following equation, respectively (weight of CUR/PSM micelles is 10.63 mg, initial weight of CUR is 1 mg, and weight of CUR in CUR/PSM micelles is 0.9149 mg).

DL (%) = (weight of CUR in CUR/PSM micelles/weight of CUR/PSM micelles) × 100%

EE (%) = (weight of CUR in CUR/PSM micelles/initial weight of CUR) × 100%

### 2.7. Stability Studies

#### 2.7.1. Stability of CUR/PSM Micelles

Evaluation of the stability of CUR/PSM micelles was carried out with EE%, DL%, particle size, and zeta potential as evaluation indicators. In brief, we stored the prepared CUR/PSM at 4 and 25 °C without light. The EE%, DL%, particle size, and zeta potential were evaluated at the corresponding time points (1, 3, 5, 7, 15, and 30 days) to study the stability of CUR/PSM micelles.

#### 2.7.2. Stability in Plasma

Notably, 2 mL of CUR/PSM micelle solution was added to 2 mL of PBS containing 10% rat plasma and incubated at 37 °C for 48 h with shaking at 100 r/min, and samples were taken at 0, 4, 6, 8, 12, 24, and 48 h. The particle size of each sample was determined by a particle size meter 3 times in parallel.

### 2.8. ROS-Responsive Drug Release

In this study, the ROS responsiveness of CUR/PSM micelles was investigated in PBS (pH 7.4) with 0 or 10 mM H_2_O_2_, which were incubated in a thermostatic shaker at 37 °C. Particle size changes were monitored by DLS at designated time points, while the micelle morphology was analyzed by TEM.

The drug release behavior of CUR/PSM micelles was studied using the dialysis method. Briefly, identical quantities of CUR solution, MSL solution, CUR/PM micelles solutions, and CUR/PSM micelles solutions were added to the dialysis bags (MWCO: 3500 Da), respectively. The dialysis bags containing CUR solution and MSL solution were positioned in 500 mL PBS (pH 7.4 and 0.5% Tween-80). In addition, the bags containing CUR/PSM micelle solutions and CUR/PM micelle solutions were placed in 500 mL of PBS (pH 7.4, containing 0.5% Tween-80) and PBS (pH 7.4, containing 0.5% Tween-80 and 10 mM H_2_O_2_) and shaken at 100 rpm at 37 °C. At 0.5, 1, 2, 4, 6, 8, 12, 24, 36, and 48 h, 3 mL of the release medium was withdrawn and analyzed using a UV-Vis spectrophotometer (8453, Agilent Ltd., Santa Clara, CA, USA) at 425 nm, followed by replenishment with an equal volume of fresh PBS.

### 2.9. Cell Uptake

For the cellular uptake study, the SW480 cells of logarithmic growth phase were seeded in a 6-well plate at a density of 2 × 10^5^ cells/well and cultured for 24 h. Then, the medium was replaced with fresh medium containing drug. After 1.5 h incubation, the cells were washed three times with PBS and then fixed with 2.5% paraformaldehyde for 15 min. Subsequently, the nucleus was stained with 4′,6-diamidino-2-phenylindole (DAPI) without light, and rinsed again with PBS for another two times. Then, uptake behavior was visualized by confocal laser scanning microscopy.

### 2.10. Cell Cytotoxicity

Cytotoxicity of blank carrier PM and PSM and drug-loaded micelles CUR/PM and CUR/PSM were evaluated using SW480 cell line by the CCK-8 method [40]. In brief, the SW480 cells of logarithmic growth phase were made into cell suspension and seeded into 96-well plates at a density of 5 × 10^3^ cells/well, then cultured for 24 h. The mediums of the micelles group were replaced with culture medium containing PM and PSM at different concentrations (0, 10, 20, 30, 50 mM) of MSL. In addition, the drug-loaded micelle groups were set to CUR + MSL (1.5:1), CUR/PM micelles and CUR/PSM micelles containing equal amounts but different concentrations (3.125, 6.25, 12.5, 25, 50, and 100 μM) of CUR. After incubation for 48 h, 10 µL CCK-8 solution was introduced into each well and incubated for another 2 h. The OD at 450 nm, representing cell viability, was measured. Cell viability was performed by the following formula:

Cell viability% = (OD_sample_ − OD_blank_)/(OD_control_ − OD_blank_) × 100%

### 2.11. Cell Migration Assay

A scratch wound healing assay was conducted to assess the migratory capacity of SW480 cells. Initially, the SW480 cells of logarithmic growth phase were plated in 6-well dishes at a density of 5 × 10^5^ cells/well and cultivated until they reached nearly full confluence. Then, a sterile 10 µL pipette tip was used to create a scratch, followed by two washes with PBS to remove cellular debris. After washing, the medium was replaced with serum-free medium. Images of the experimental and control groups were captured at both the initial time point (0 h) and after 48 h using an inverted microscope.

### 2.12. Preparation and Characterization of KGM-CUR/PSM Microspheres

#### 2.12.1. Preparation of KGM-CUR/PSM Microspheres

KGM-CUR/PSM microspheres were prepared by emulsification cross-linking method. Firstly, 0.05 g CUR/PSM was dissolved in 4% KGM solution as aqueous phase (W), and then Span-80 emulsifier was added to liquid paraffin as oil phase (O). The KGM solution was slowly added into the oil phase with a syringe and stirred at 50 °C for 30 min to form a stable W/O emulsion. The mixed solution of CUR/PSM and KGM formed tiny droplets in the oil phase. Notably, 0.5 mL of 2% glutaraldehyde was added as a cross-linking agent. After 2 h of stirring and curing, the droplets were crosslinked into microspheres. The solid was obtained by centrifugal filtration and washed quickly with petroleum ether and purified water. Finally, KGM-CUR/PSM microspheres were obtained by vacuum drying.

#### 2.12.2. Characterization of KGM-CUR/PSM Microspheres

The morphology of the KGM-CUR/PSM microspheres was examined using Regulus 810 Field Emission Scanning Electron Microscopy (Hitachi Science Instruments Ltd., BeiJing, China). The particle size was measured using dynamic light scattering at room temperature (DLS, Nano-s, Malvern Instruments Ltd., Malvern, UK). The Fourier-Transform Infrared (FT-IR) spectra of KGM-CUR/PSM microspheres were recorded using a Spectrum 100 FT-IR spectrometer (PerkinElmer Ltd., Shanghai, China). The thermal properties of CUR/PSM micelles, KGM, physical mixtures of CUR/PSM + KGM, and KGM-CUR/PSM microspheres were analyzed by STA409PC thermogravimetric analyzer. (DSC, STA409PC, Hengjiu Ltd., Xuzhou, China)

The drug loading (DL%) and encapsulation efficiency (EE%) of the KGM-CUR/PSM microspheres were assayed at 425 nm by HPLC. (HPLC conditions were the same as those described in Section 2.6). DL% and EE% of the CUR in KGM-CUR/PSM microspheres were assessed by the following formulas:

DL% = (weight of CUR in micelles/weight of KGM-CUR/PSM) × 100%

EE% = (weight of CUR in micelles/weight of CUR added during preparation) × 100%.

### 2.13. ROS/Enzyme Dual-Responsive Drug Release

The controlled-release characteristic and ROS and enzyme dual-sensitivity of KGM-CUR/PSM microspheres were investigated by simulating in vitro release environments (colorectal microenvironment and tumor microenvironment), and the in vitro release profiles of the drug-loaded system were measured to investigate the release characteristics of KGM-CUR/PSM microspheres under different conditions, so as to evaluate the dual-targeting properties of KGM-CUR/PSM microspheres with respect to ROS and enzyme. In brief, the mixed solution of 20 mg KGM-CUR/PSM microspheres and 2 mL of PBS (pH 7.4) was put in dialysis bag, which was immersed in artificial gastric fluid (pH 1.2) at 37 °C and horizontally shaken for 2 h at a speed of 100 rpm. Afterwards, the release medium was sequentially adjusted to artificial small bowel fluid (pH 6.8) and artificial colon fluid (pH 7.4 PBS solution + 0.5% Tween-80 + 10 mM H_2_O_2_ + 0.2 U/mL β-mannanase) and incubated for 3 h and 31 h, respectively. At predetermined time points, 3 mL of the release medium was removed and replenished with an equivalent amount of fresh solution. After passing through the membrane, the peak areas of CUR and MSL were analyzed by HPLC. (The HPLC conditions for CUR were as described in Section 2.6; for MSL: column: Welch Materials (4.6 × 250 mm, 5 μm); mobile phase: phosphate buffer (pH 6.8)-methanol (50:50, *v*/*v*); flow rate: 1.0 mL·min^−1^; column temperature: 25 °C; detection wavelength: 303 nm; injection volume: 10 μL). In addition, the cumulative release rate was determined, and the release profile of KGM-CUR/PSM microspheres was plotted.

### 2.14. Animal Experiments and Design

The male C57BL/6 mice were housed for 7 days at a temperature of 23 ± 2 °C and an average humidity of 58 ± 5 °C and all mice were provided access to food and water freely. Subsequently, mice were randomly assigned to seven groups (twelve mice per group): the control group, model group, KGM group, CUR + MSL group, CUR/PM micelles group, CUR/PSM micelles group, and KGM-CUR/PSM microspheres group.

Hemolysis experiment was conducted to evaluate the hemocompatibility of KGM-CUR/PSM microspheres. Mouse orbital blood was collected into heparin-coated centrifuge tubes and centrifuged (10 min, 1000× *g*). The erythrocytes were washed with physiological saline until the supernatant was colorless. A 2% erythrocyte suspension was prepared by mixing 200 µL of packed erythrocytes with 10 mL of physiological saline. For the assay, 1 mL of the 2% erythrocyte suspension was aliquoted into centrifuge tubes, followed by the addition of 3 mL of KGM-CUR/PSM solutions at concentrations of 1, 2, 4, 8, and 16 mg/kg, respectively. Negative controls received 3 mL of physiological saline, while positive controls were treated with 3 mL of distilled water. All test groups were incubated at 37 °C for 4 h, then centrifuged (4 °C, 3000 r/min, 10 min). The absorbance of the supernatant from each group was measured at 450 nm. The hemolysis percentage was calculated using the following formula:

Hemolysis rate (%) = (*A*_sample_ − *A*_negative_)/(*A*_positive_ − *A*_negative_) × 100%

Azoxymethane (AOM), a genotoxic colon carcinogen, and dextran sodium sulfate (DSS), an inflammatory agent that induces colitis, were used to establish the CRC mouse model. Except for the normal group, all other groups were intraperitoneally administered AOM (10.0 mg/kg body weight) on Day 1. From Day 8, mice received 2% (*w*/*v*) DSS solution for 7 days followed by 14 days of normal drinking water, with this cycle repeated three times concluding on Day 70. (Successful colorectal cancer induction was confirmed by four criteria: (i) >15% body weight reduction, (ii) diarrhea or loose stools, (iii) fecal occult blood positivity, and (iv) the presence of CRC tumors was further confirmed in preexperiments by sacrificing mice that developed the above symptoms.) From day 71 onward, the treatment groups (KGM group, CUR + MSL group, CUR/PM micelles group, CUR/PSM micelles group, and KGM-CUR/PSM microspheres group) were administered corresponding drugs via oral gavage at a dose of 100 mg/kg every two days for 2 weeks. The control group and the model group received an equivalent volume of distilled water via oral gavage. On Day 84, after 12 h fasting, mice were sacrificed by cervical dislocation with immediate collection of colon tissues and orbital blood for subsequent analysis. Daily records included food intake, body weight, mental status, fecal consistency, and anal alterations throughout the 84-day study.

### 2.15. Histology Analysis

For histopathological analysis, the colon tissues were placed in 4% formaldehyde solution, dehydrated, and impregnated with paraffin. Then, the tissues were sectioned and stained with hematoxylin and eosin (H&E), followed by observation under a light microscope.

### 2.16. Inflammation Analysis

#### 2.16.1. Collection of Serum Samples and Expression Levels of TNF-α, IL-1β, IL-6 in Mice

After 16 h of model building, the blood was obtained from eyeballs of mice. The blood samples were placed in centrifuge tubes and kept standing at 4 °C for 1 h, and then centrifuged in a centrifuge (4 °C, 12,000 rpm) for 5 min. The expression levels of IL-1β, IL-6, and TNF-α in serum were detected by an ELISA assay kit according to the instructions (Ebioscience, San Diego, CA, USA). The OD was determined by microplate reader at 450 nm.

#### 2.16.2. Detection of TNF-α, IL-1β and IL-6 Expression Levels in Colorectal Tissues

About 0.1 g of colon tissue was cut and added to 0.5 mL of pre-cooled PBS solution for homogenization, and then centrifuged at 15,000 rpm for 15 min to obtain the supernatant. According to the ELISA kit instructions, the 96-well plate containing the samples was placed in the enzyme labeler to test the OD of each hole at 450 nm.

#### 2.16.3. Determination of MPO Expression Levels in CRC Mice

Myeloperoxidase (MPO) is a specific marker of neutrophils in the mouse colon tissue. The colorectal tissues of mice were taken and weighed, then 9 times the volume of PBS solution was added, the colon tissues were fully homogenized with a homogenizer, and the supernatant was collected after centrifugation for 10 min.

### 2.17. Microbiological Analysis of Intestinal Microbiota

Feces were collected from the colorectal contents of mice under aseptic conditions using sterile freezing tubes and stored at −80 °C. The fecal genomic DNA was extracted using a Stool DNA Kit (Bemac Biotech Co., Ltd., Nanjing, China) according to the manufacturer’s experimental steps. Then, the 16S rRNA V3-V4 hypervariable region was amplified by PCR using barcoded primers (341F/806R), and the amplicons were purified and collected under thermal cycling conditions. Sequencing was performed after quantification with a Qubit dsDNA HS Assay Kit. Subsequently, OTU analysis, Alpha diversity analysis, and Beta diversity analysis were conducted through QIIME software (version 1.9.1), and the abundance of intestinal microbiota in each group was analyzed with OTU taxonomic results.

### 2.18. Statistical Analysis

Statistical analyses were performed using Graph Pad Prism 9.0 software. All results were expressed as “mean ± SD”. Comparisons between groups were analyzed by one-way ANOVA. Additionally, t-test method was used for comparison between two groups. *p* < 0.05 was considered statistically significant.

## 3. Results

### 3.1. Synthesis and Characterization of PSM and PM

#### 3.1.1. Synthesis of PSM and PM

**Figure 2:**
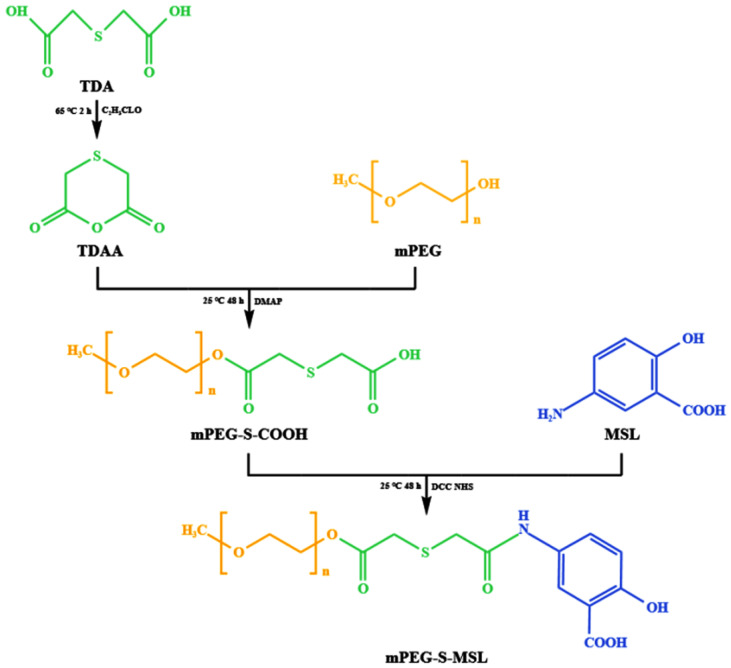
Synthesis process of PSM. (Orange (mPEG): represents the hydrophilic polyethylene glycol monomethyl ether segment; Green (TDA and TDAA): indicates the thioether linker (2,2′-thiodiacetic acid and its anhydride); Blue (MSL): Denotes the anti-inflammatory drug mesalazine, serving as hydrophobic segment.

**Figure 3:**
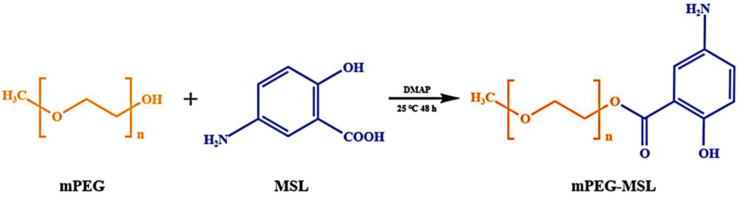
Synthesis process of PM. (Orange (mPEG): Hydrophilic polyethylene glycol monomethyl ether segment; Blue (MSL): Mesalazine directly conjugated via ester bond.

To synthesize PSM and PM, Figure 2 shows the synthetic route of PSM. Firstly, TDA was dehydrated and condensed to form the anhydride TDAA. Then, mPEG-S-COOH was formed from TDAA and mPEG via an esterification reaction. Finally, mPEG-S-COOH was reacted with MSL via amidation to yield mPEG-S-MSL (PSM) using DCC and NHS as catalysts, respectively. In addition, mPEG and MSL were reacted by esterification to produce mPEG-MSL (PM), as shown in Figure 3.

#### 3.1.2. Proton Nuclear Magnetic Resonance (^1^H-NMR) and Mass Spectrometry (MS) Analysis

**Figure 4:**
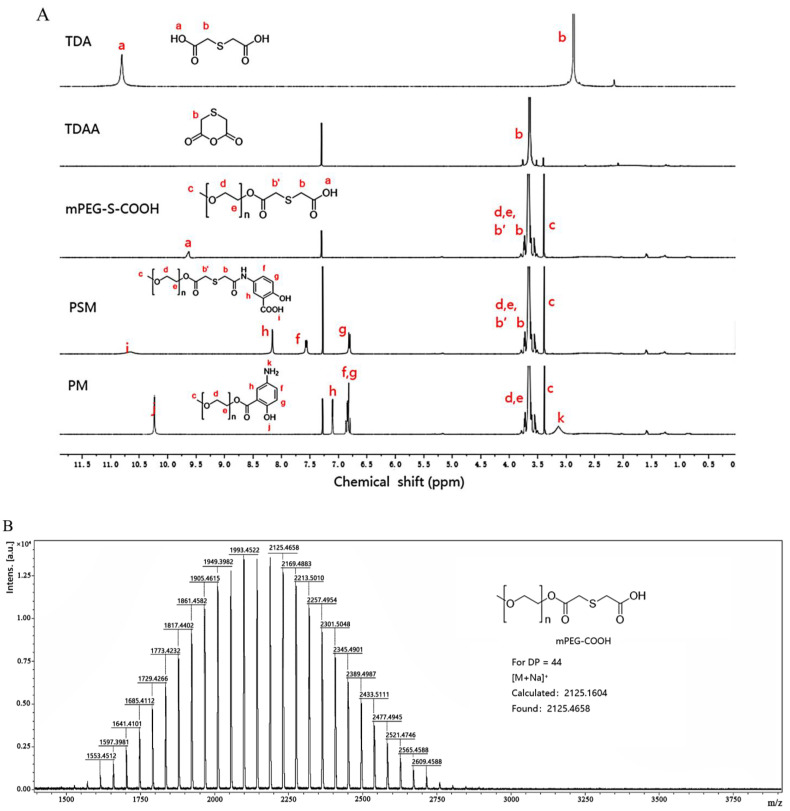

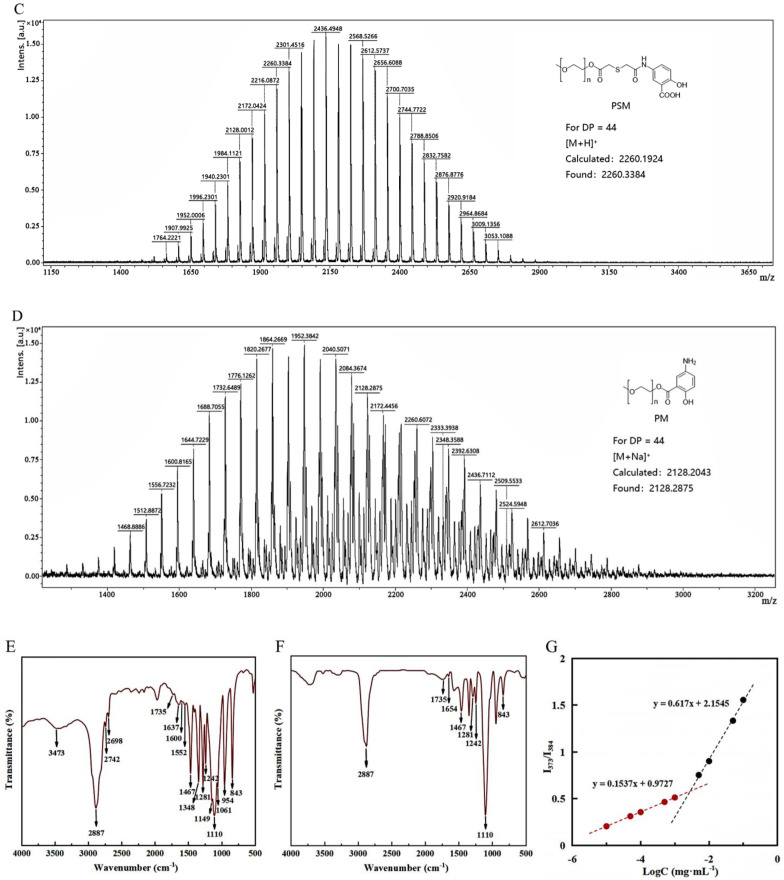
The ^1^H NMR spectra of TDA, TDAA, mPEG-S-COOH, PSM, and PM (TDA, TDAA, mPEG-S-COOH, PSM, MSL, and PM were dissolved in chloroform and determined by 400 MHz ^1^H-NMR spectrometry) (**A**). The mass spectrometry analysis spectra of mPEG-S-COOH (**B**), PSM (**C**), and PM (**D**). FT-IR spectra of PSM (**E**) and PM (**F**). Plot of the fluorescence intensity ratio (I_373_/I_384_) against the logarithm of PSM concentration (**G**).

The ^1^H-NMR spectra of PSM and PM polymers were depicted in Figure 4A. For TDA, the peaks that appeared at 12.35 and 3.64 ppm were attributed to the carboxyl group (-COOH) and -CH_2_-S-CH_2_- group in TDA. Compared with TDA, the typical peak of -COOH (12.35 ppm) disappeared and the -CH_2_-S-CH_2_- group remained unchanged at 3.64 ppm. These showed that TDAA was successfully synthesized. In the ^1^H NMR spectrum of mPEG-S-COOH, the characteristic peaks at 3.3, 3.5–3.8, and 9.7 ppm belonged to the -CH_3_, -CH_2_-, and -COOH, respectively. In addition, the theoretical [M + Na]^+^ value of mPEG-S-COOH was calculated to be 2125.1604, which is consistent with the observed mass spectrometry peak at *m*/*z* 2125.4658, confirming the successful synthesis of mPEG-S-COOH. For PSM, the peak that appeared at 10.6 ppm was assigned to the -COOH, phenolic hydroxyl group of MSL. The phenolic appeared at 8.2 ppm and the signals at 7.6 and 6.8 ppm were due to the characteristic peak of the benzene ring of MSL. Then, there was a characteristic peak of the -CH_3_ at 3.3 ppm. Furthermore, mass spectrometric analysis showed excellent agreement between the observed [M + Na]^+^ peak (*m*/*z* 2260.3384) and the theoretical value (2260.1924), confirming the successful synthesis of PSM. In the ^1^H NMR spectrum of PM, the signals for the -OH, the phenolic hydroxyl group of MSL, the -CH_3,_ and the -NH- appeared at 10.2, 6.7–7.1, 3.3, and 3.1 ppm, respectively. Moreover, mass spectrometry analysis revealed an [M + Na]^+^ peak at *m*/*z* 2128.2875, in excellent agreement with the theoretical value (2128.2043), confirming the successful synthesis.

#### 3.1.3. Fourier-Transform Infrared (FT-IR) Spectroscopy Analysis

In addition, the PSM structure was confirmed by FT-IR (Figure 4E). The broad peak at 3473 cm^−1^ was due to the hydroxyl group and the overlapping elastic vibration peaks of the N-H in MSL amino groups. The peak that appeared at 2887 cm^−1^ was the stretching vibration peak of the methylidene (-CH_2_-) in the mPEG fatty chains. There were distinct peaks at 1281 and 1242 cm^−1^, which were assigned to the ether bond (C-O-C) in mPEG stretching vibration. The two sharp peaks that appeared at 2742 and 2698 cm^−1^ were the stretching vibration peaks of the methylene (-CH-) in TDAA. The peaks at 1149, 1110, and 1061 cm^−1^ indicated the presence of ester bonds (-COOC-). Moreover, the peaks that appeared at 1735, 1467, and 843 cm^−1^ were attributed to the absorption peaks of the carbonyl group (-C=O) in the ester bond, benzene ring (C=C), and ortho-disubstituted benzene (=C-H), respectively. The peaks of the carbonyl group (-C=O) bending vibration at 1637 (amide I band), 1552 (amide II band), and 1348 cm^−1^ (amide III band) were observed. Therefore, the above data further proved the successful synthesis of the mPEG-S-MSL polymer.

For PM (Figure 4F), the typical peaks at 2887 and 1242 cm^−1^ were due to the methylidene (-CH_2_-) and the ether bond (C-O-C) in mPEG, while the peaks at 1467 and 843 cm^−1^ were the characteristic peaks of the benzene ring of MSL. In addition, the presence of the stretching vibration peak of the carbonyl groups (-C=O) on ester bonds at 1735 cm^−1^, proved that the hydroxyl group (-OH) in mPEG was successfully connected to the carboxyl group (-COOH) in MSL. The results suggested that the PM had been successfully synthesized.

### 3.2. Determination of the Critical Aggregation Concentration (CAC)

The intensity ratio (I_373_/I_384_) of the pyrene excitation spectra versus the logarithm of copolymer concentration is shown in Figure 4D. At minimal concentrations of the copolymer, the overall fluorescence intensity ratio remained virtually constant. When the copolymer concentration reached the CAC, the intensity ratio began to increase greatly, the concentration of the copolymer increased, and the intensity ratio began to stabilize gradually. These results suggested a shift in the characteristics of pyrene from an aqueous environment to a hydrophobic one. According to the intersection of the two straight lines, the CAC of PSM was determined as 2.81 × 10^−3^ mg·mL^−1^, which illustrated that PSM would exhibit good thermodynamic stability under dilution conditions.

### 3.3. CUR and MSL Synergistically Inhibit the Proliferation of SW480 Cells

**Figure 5:**
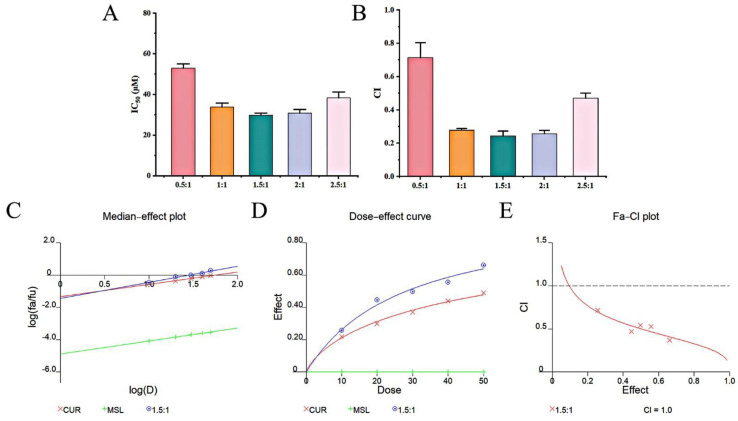
IC50 values (**A**) and CI values (**B**) for different proportions of CUR and MSL, median–effect plot (**C**), dose–effect curve (**D**), and Fa-CI plot (**E**) of CUR and MSL. The dotted line indicates the threshold for Combination Index (CI) values, where CI < 1 signifies synergy, CI = 1 indicates additive effects, and CI > 1 represents antagonism between CUR and MSL.

The CCK-8 assay and the median–effect principle (Chou-Talalay combined index method) were used to evaluate the synergistic effect of CUR and MSL. As summarized in Table 2, the inhibition rates of SW480 cells were determined at total drug concentrations of 10–50 μM for both single-agent (CUR or MSL alone) and combination groups (CUR:MSL molar ratios from 0.5:1 to 2.5:1). Then, as shown in Figure 5A–E, the IC_50_ values, CI values, median–effect curve, dose–effect curve, and Fa-CI curve were obtained by Calcusyn software. The median–effect curve and dose–effect curve showed that the treatment effect of the combination of CUR and MSL was more effective than CUR or MSL alone. The Fa-CI curves showed that CI < 1 at different ratios, indicating that the CUR and MSL showed a synergistic effect. When the CUR:MSL was between 1:1 and 2:1, the CI value was less than 0.3, which further indicated that the combined drug delivery system displayed a strong synergistic effect. Therefore, the follow-up experiments were selected based on the ratio of CUR and MSL within the optimal range of synergistic ratio (1:1~2:1).

### 3.4. Preparation and Characterization of CUR/PSM Micelles

**Figure 6:**
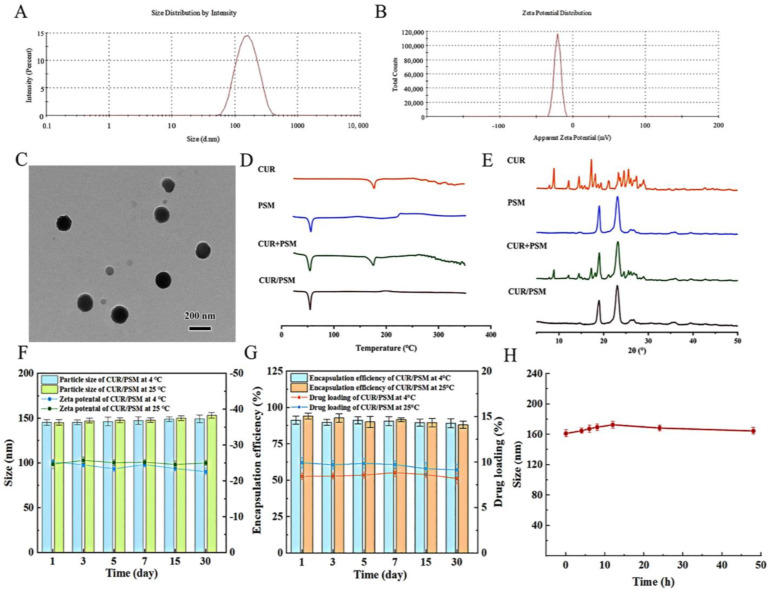
Dynamic light scattering particle size distribution (**A**) and zeta potential (**B**) of the CUR/PSM micelles. Transmission electron micrograph of CUR/PSM micelles (**C**). DSC profiles of CUR/PSM micelles (**D**). XRD spectra of CUR/PSM micelles (**E**). Particle size distribution and zeta potential of CUR/PSM micelles (**F**), EE% and DL% of CUR/PSM micelles (**G**), and stability in plasma of CUR/PSM micelles (*n* = 3) (**H**).

The particle size, polydispersity index (PDI), and zeta potential of CUR/PSM micelles were studied by DLS, and the results are presented in Figure 6A,B. The mean particle size, PDI, and zeta potential of CUR/PSM micelles were 144.45 ± 1.56 nm, 0.123 ± 0.02, and −25.01 ± 0.11 mV, respectively. The HPLC results showed that the DL% and EE% of CUR/PSM micelles were 8.61 ± 0.08% and 91.49 ± 0.26%, respectively.

The morphological characterization of CUR/PSM micelles was observed by TEM, which showed that CUR/PSM micelles were spherical with smooth edges and uniform in particle size (Figure 6C), indicating that the prepared CUR/PSM micelles were well dispersed.

The DSC scans of CUR, PSM, CUR + PSM, and CUR/PSM micelles are shown in Figure 6D. The CUR showed an obvious endothermic peak at 179.6 °C, and a clear endothermic peak appeared in PSM at 179.6 °C. For the CUR + PSM, the characteristic peak of CUR was still present in the spectrum. In contrast, the endothermic peak of CUR disappeared in the CUR/PSM micelles spectrum, which suggested that CUR was fully encapsulated within the hydrophobic core in a disordered state, rather than adhering to the micelle surface.

The XRD patterns of CUR, PSM, CUR + PSM, and CUR/PSM micelles are shown in Figure 6E. The image of CUR displayed absorption peaks at 8.88°, 12.19°, 14.49°, 17.25°, 18.13°, 21.15°, 23.72°, 24.57°, 28.14, and 28.95°. The blank carrier of PSM showed two strong absorption peaks at 19.02° and 23.11°. For their physical mixture, CUR + PSM. Additionally, the spectra of CUR + PSM showed distinctive absorption peaks of CUR and PSM blank carriers, while the CUR/PSM spectra only displayed two obvious absorption peaks at 19.02° and 23.11°, and the absorption peaks of CUR disappeared, which further showed that the CUR was encapsulated inside the micelles in an amorphous state.

Stability is an important factor in evaluating micelle behavior. As shown in Figure 6F, the particle size and zeta potential were not significantly changed during the storage time. In addition, there were no significant variations in EE% and DL% (Figure 6G). Collectively, these results indicated that the CUR/PSM micelles were very stable. Figure 6H demonstrated a slight initial increase in the particle size of the CUR/PSM micelles, followed by a stable plateau over a 48 h period, suggesting that the CUR/PSM micelles exhibited excellent plasma stability.

### 3.5. ROS-Responsive Drug Release

**Figure 7:**
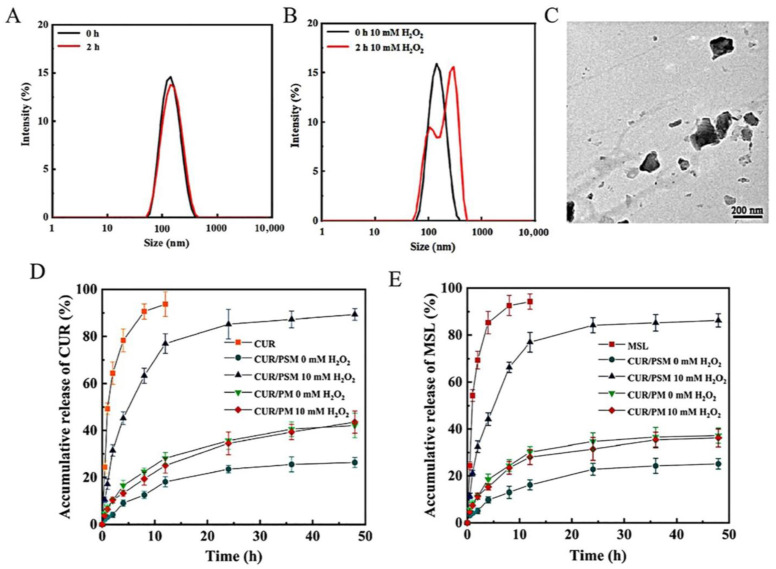
Particle size distribution of CUR/PSM micelles at different time points in 0 (**A**) and 100 (**B**) mM H_2_O_2_. Transmission electron micrograph of CUR/PSM micelles in 10 mM H_2_O_2_ (**C**). Drug release profiles of CUR (**D**) and MSL (**E**) in CUR/PSM micelles at 0.5, 1, 2, 4, 6, 8, 12, 24, 36, and 48 h (*n* = 3).

As shown in Figure 7A,B, the ROS responsiveness of CUR/PSM micelles was evaluated by observing the time-dependent variation in micelle sizes in response to ROS. The DLS results indicated that the particle size of CUR/PSM micelles remained relatively constant in the absence of H_2_O_2_. However, the particle size became larger and unevenly distributed in the presence of 10 mM H_2_O_2_, which suggested that the carriers had responsively disintegrated in the oxidizing environment. As shown in the TEM images (Figure 7C), the core-shell structure of CUR/PSM micelles was disrupted into irregular nano-aggregates after incubation with 10 mM H_2_O_2_ for 4 h. These results confirmed that CUR/PSM micelles were highly sensitive to ROS.

In addition, the ROS-responsive drug release behavior of CUR/PSM micelles was studied using the dialysis bag mean at PBS (pH 7.4) containing 0 or 10 mM H_2_O_2_ to simulate the release of CUR and MSL in SW480 cells, respectively. As shown in Figure 7D,E, the free CUR and MSL were almost completely released within 12 h, while the CUR/PSM micelles and CUR/PM micelles significantly delayed the rate of drug release. H_2_O_2_ showed no significant effect on the release of CUR/PM micelles, demonstrating that CUR/PM micelles showed no ROS responsiveness. However, the cumulative release rates of CUR and MSL from CUR/PSM micelles in H_2_O_2_-free medium were only 26.38 ± 2.14% and 25.17 ± 2.26%, respectively. The reason for this difference was that the sulfide-ether and amide bonds in the carrier structure increased the stability of CUR/PSM micelles. The cumulative release rates of CUR and MSL from CUR/PSM micelles in H_2_O_2_-containing medium were 89.32 ± 2.48% and 86.25 ± 2.75%, respectively, which further suggested that CUR/PSM micelles showed a high sensitivity to ROS.

### 3.6. Cell Uptake

**Figure 8:**
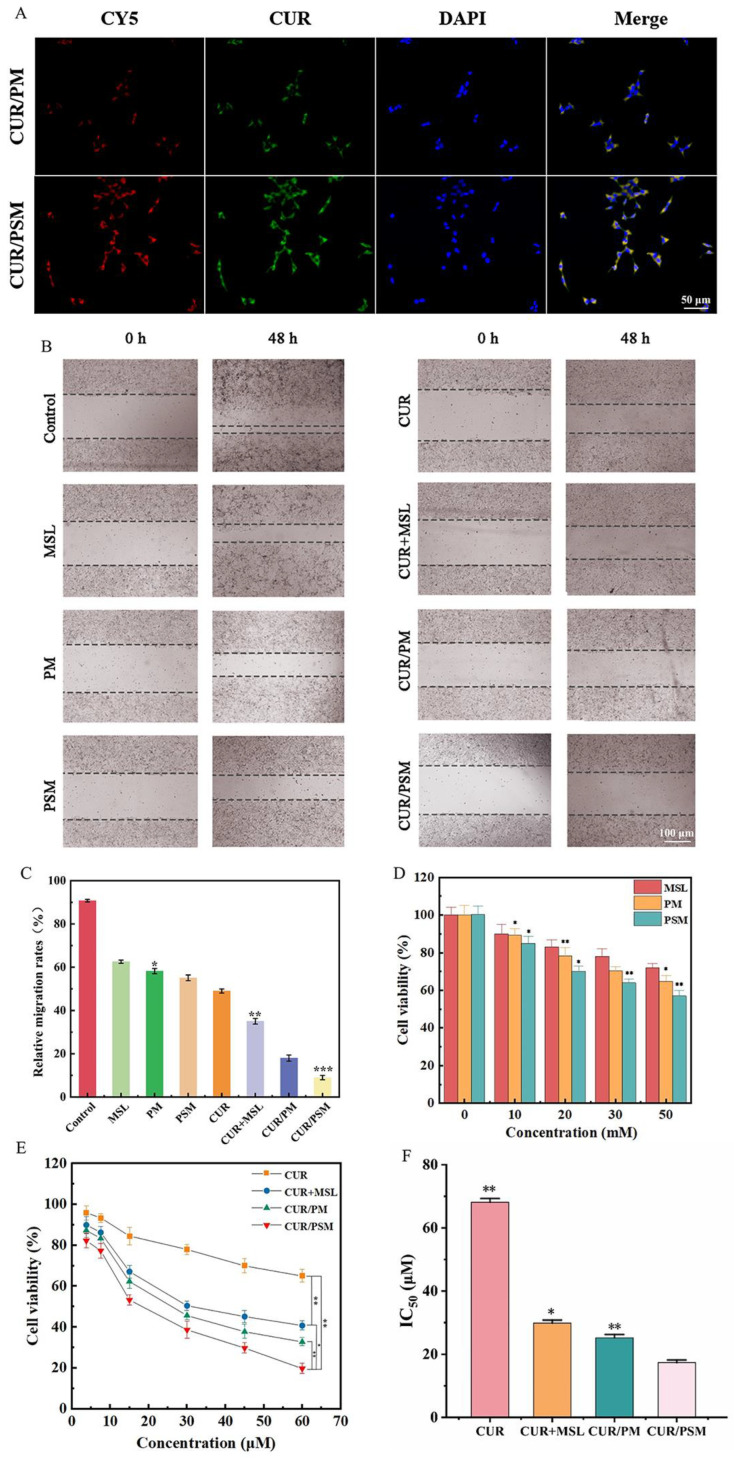
Cellular uptake of loaded CUR micelles in SW480 cells (**A**). Effects of different treated groups on migration of SW480 cells (CUR concentration: 50 pg/mL). (scale bars: 100 μm) (**B**) and relative migration rates (**C**) (*n* = 3, compared with control, * *p* < 0.05, ** *p* < 0.01, *** *p* < 0.001). Effects of cells survival rate of MSL, PM, and PSM on SW480 cells (**D**) (*n* = 3, compared with MSL, * *p* < 0.05, ** *p* < 0.01). Cell viability rate using different CUR formulations after 48 h (**E**). (*n* = 3, compared with CUR/PSM, * *p* < 0.05, ** *p* < 0.01). IC_50_ values of different drug groups (**F**). (*n* = 3, compared with CUR/PSM, * *p* < 0.05, ** *p* < 0.01).

Effective cellular uptake was crucial for assessing the uptake ability of cells (Figure 8A). Since MSL was not detectable under a fluorescence inverted microscope, CY5 was utilized as a fluorescent marker to substitute for MSL in this experiment. As illustrated in Figure 8A, both SW480 cells treated with CUR/PSM micelles and CUR/PM micelles exhibited red fluorescence from CY5, green fluorescence from CUR, and blue nuclear fluorescence, which indicated that MSL and CUR could be released intracellularly. Differently, the CUR/PSM micelles group displayed a higher fluorescence intensity in the cytoplasm than the CUR/PM micelles group. This could be attributed to CUR/PSM micelles possessing a remarkable ROS-responsive release ability which promoted greater drug release.

### 3.7. Cell Migration Assay

The migration of SW480 cells was observed by a scratch wound healing test, and the relative migration rates were calculated by ImageJ software (Version 2.0; National Institutes of Health, USA) (Figure 8B,C). After 48 h of incubation, the drug-treated scratches all showed different degrees of healing compared to the untreated blank control group. Simultaneously, CUR/PSM micelles displayed the strongest inhibitory effect on SW480 cell migration at 48 h compared with the other groups, which was significant for inhibiting tumor metastasis.

### 3.8. Cell Cytotoxicity

The cytotoxicity of the blank carrier (PM and PSM) and drug-loaded micelles group (CUR + MSL, CUR/PM micelles, and CUR/PSM micelles) to cells was evaluated by the CCK-8 method. In the blank carrier group, the cell viability of each group decreased with the increase in MSL concentration. Compared with MSL and PM, PSM showed stronger cytotoxicity. This suggested that in the presence of ROS, PSM increased the enrichment of MSL in SW480 cells (Figure 8C). Additionally, the inhibition of CUR, CUR + MSL, CUR/PM micelles, and CUR/PSM micelles on SW480 cells was summarized in Figure 8D, which showed that with a rise in CUR concentration, the cell viability of each group gradually decreased. Meanwhile, compared with CUR alone, the CUR + MSL, CUR/PM micelles, and CUR/PSM micelles significantly increased the inhibition rate, suggesting that MSL as an anti-inflammatory agent could enhance the efficacy of CUR by modulating inflammatory factors in the tumor microenvironment. The IC_50_ values of each drug-loaded micelle group on SW480 cells after 48 h are shown in Figure 8E. CUR/PSM micelles showed the highest cytotoxicity to SW480 cells at an equivalent CUR dose. The above results further indicated that the high concentration of ROS in the SW480 cells stimulated the breakage of the thioether bond in the CUR/PSM micelles to cause a higher release of the drug that produced more toxicity to the SW480 cells.

### 3.9. Preparation and Characterization of KGM-CUR/PSM Microspheres

Aiming at the complex environment of CRC, and with the goal of further achieving precise targeted treatment of CRC, KGM was used to cover CUR/PSM by the emulsification cross-linking method to prepare KGM-CUR/PSM microspheres, and a ROS/enzyme dual-responsive drug delivery system was established.

Based on the aforementioned calculation approach, the DL% and EE% of the CUR in KGM-CUR/PSM microspheres reached up to 1.63 ± 0.04% and 68.56 ± 1.06%, respectively. Both the encapsulation efficiency and drug loading were reduced, which may be caused by the increase in carrier materials.

**Figure 9:**
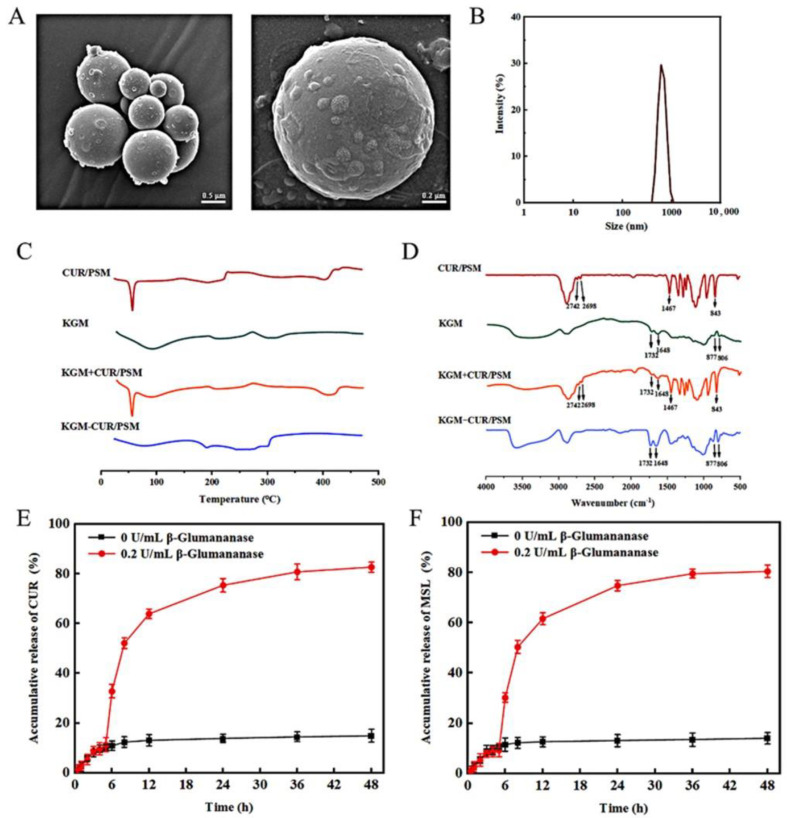
SEM plot of KGM-CUR/PSM microspheres (**A**). Particle size distribution of KGM-CUR/PSM microspheres (**B**). DSC profiles of KGM-CUR/PSM microspheres (**C**). FT-IR spectra of KGM-CUR/PSM microspheres (**D**). Cumulative drug release curves of KGM-CUR/PSM microspheres, CUR (**E**) MSL (**F**) (*n* = 3). Notably, 0–2 h: artificial gastric fluid (pH 1.2); 2–5 h: artificial small bowel fluid (pH 6.8); 5–36 h: artificial colon fluid (pH 7.4 PBS solution + 0.5% Tween-80 + 10 mM H_2_O_2_ + 0.2 U/mL β-mannanase).

The surface morphologies of the KGM-CUR/PSM microspheres were visualized using SEM (Figure 9A). The KGM-CUR/PSM microspheres were regular and spherical in shape with no aggregation, and they possessed homogeneous size and good dispersion. The mean particle size of KGM-CUR/PSM microspheres was 1.03 ± 0.05 μm (Figure 9B). Figure 9C shows DSC scans of CUR/PSM micelles, KGM, CUR/PSM + KGM, and KGM-CUR/PSM microspheres. An endothermic peak was noted at 54.6 °C for CUR/PSM micelles and this characteristic peak was still present in CUR + PSM. However, for KGM-CUR/PSM microspheres, the characteristic peak of CUR/PSM micelles disappeared, which indicated that CUR/PSM micelles were completely encapsulated in KGM-CUR/PSM microspheres in an amorphous state.

Figure 9D shows the FT-IR spectra over the range of 500–4000 cm^−1^ for CUR/PSM micelles, KGM, physical mixtures, CUR/PSM + KGM, and KGM-CUR/PSM microspheres. For KGM, the characteristic absorption peak of the -OH group was observed at 4000–2500 cm^−1^. The peak observed at 2930 cm^−1^ was attributed to the presence of the C-H group stretching vibration. The peak at 1732 cm^−1^ was assigned to the C=O peak of the acetyl group. The peak at 1648 cm^−1^ was generated by hydrogen bonding within the polysaccharide molecule. The absorption peak at 877 cm^−1^ appeared in the β-D glycosidic bond configuration, and the signal appearing at 806 cm^−1^ was related to the pyran ring. These results indicated that mannose was the basic component of mannan. These peaks were found in both KGM, KGM + CUR/PSM, and KGM-CUR/PSM microspheres but not in CUR/PSM micelles. Similarly, the characteristic peaks of the benzene ring, amide group, and ester group in CUR/PSM micelles were absent in KGM-CUR/PSM microspheres, but appeared in KGM + CUR/PSM. These results suggested that CUR/PSM micelles were successfully encapsulated in KGM-CUR/PSM microspheres.

As shown in Figure 9E,F, the drug release rate was less than 20% in pH 1.2 gastric fluid and pH 6.8 intestinal fluid (both with H_2_O_2_ but no enzyme) in 1 to 5 h, which suggested that CUR/PSM micelles were protected by encapsulation in KGM. The cumulative release rates of CUR and MSL were 81.67% and 80.45% in pH 7.4 artificial colonic fluid containing 10 U/mL β-mannanase and 10 mM H_2_O_2_, respectively. The result showed that KGM-CUR/PSM microspheres would not be released under oxidative and acidic conditions in the stomach and small intestine, but relied on the joint action of the enzyme and ROS. KGM could be hydrolyzed into small molecules such as gluconic acid, monosaccharides, and disaccharides under the action of β-mannanase in the colorectum, thereby exposing CUR/PSM micelles and releasing the drug under the condition of ROS, which could achieve the effect of precise localization of drug release in CRC.

### 3.10. Animal Experiments and Designed

**Figure 10:**
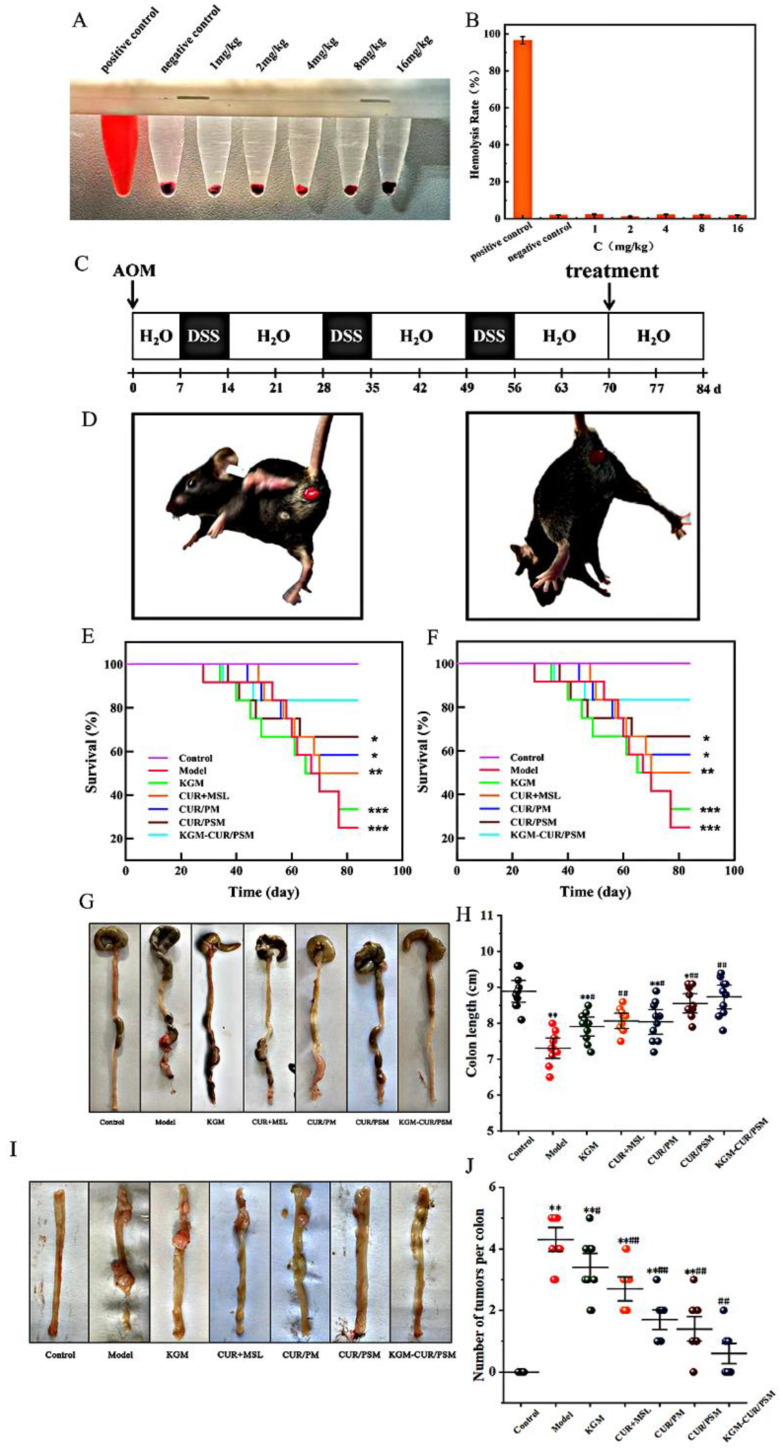
Hemolysis assay: visual samples (**A**) and quantitative hemolysis rates (**B**). Establishment process of the CRC mouse model (**C**). Appearance of CRC mice during the dosing intervention (**D**), body weight growth curve (**E**) (*n* = 12, compared with control, * *p* < 0.05, ** *p* < 0.01, *** *p* < 0.001), and survival rate (**F**) during the molding of CRC mice (*n* = 12, compared with control, * *p* < 0.05, ** *p* < 0.01, *** *p* < 0.001). Appearance (**G**), length distribution (**H**), physical tumor (**I**), and distribution of tumor number (**J**) of CRC mice (*n* = 12, compared with control, ** *p* < 0.01; compared with model, # *p* < 0.05, ## *p* < 0.01).

As shown in Figure 10A,B, the positive control group (distilled water) exhibited a clear red solution due to massive hemolysis caused by erythrocyte rupture from water absorption. In contrast, all KGM-CUR/PSM solutions at different concentrations demonstrated erythrocyte sedimentation with clear and transparent supernatants. The hemolysis rates were consistently below 3%, which complied with pharmacopeial standards. These results indicate negligible hemolytic activity and excellent biosafety of the tested formulations.

We successfully constructed the AOM/DSS-induced colorectal cancer mice model according to the experimental design in Figure 10C. An animal model of inflammatory colorectal cancer was established by AOM/DSS combined induction in mice. During the modeling period, the weight of the mice in the control group increased, while there was no significant change in other characteristics. All the other groups lost weight after AOM injection and then gained weight. At the initial stage of DSS administration, the mice in each group showed symptoms of colitis, including diarrhea, lethargy, loss of appetite, weight loss, etc., and mucus stools and bloody stools gradually appeared at a later stage, which were relieved after normal water intake. After the administration of the second cycle of DSS, the mice began to show a certain DSS tolerance, and the symptomatic manifestation was slightly less than that in the first cycle. Moreover, prior to treatment initiation during the third DSS cycle, all mice in the AOM/DSS-induced group exhibited markedly severe clinical symptoms of CRC, including pronounced emaciation, significant body weight loss, increased hematochezia, tachypnea (rapid breathing), and convulsions, accompanied by a substantial decline in survival rate. These collective observations confirm the successful establishment of a severe CRC phenotype. During the dosing intervention, some of the mice in the model group developed severe prolapse due to untreated mice (Figure 10D), while the other groups had slightly different degrees of reduction in symptoms compared to the model group. In contrast, the KGM-CUR/PSM microsphere group showed the fastest weight recovery and a significantly higher survival rate (Figure 10E,F).

After the mice were sacrificed at the end of the modeling, the colorectum was removed along the anus to the ileocecal region, as shown in Figure 10G–J. The colon of the control group was smooth without tumor, and the length ranged from 8.1 cm to 9.7 cm. In the model group, the colon length was 6.5–8.1 cm and the number of tumors was over 4. Meanwhile, we could observe the thinning of the colonic mucosa and congestion in the intestine. KGM, CUR + PSM, CUR/PM micelles, and CUR/PSM micelle groups all showed different improvements compared with the model group. The number of tumors ranged from 1 to 4 and the tumor size was relatively small. Compared with the model group, the number and volume of tumors all decreased in the KGM, CUR + PSM, CUR/PM micelles, and CUR/PSM micelle groups. The CUR + MSL group showed poor tumor suppression. Tumor sizes were decreased in the CUR/PM micelles group, but some visible tumors were still present. CUR/PSM micelles significantly inhibited tumor growth and reduced tumor size, but did not completely remove the tumor. As expected, the KGM-CUR/PSM microspheres group showed the most optimal therapeutic effect. For example, the length of the colon was 8~9.5 cm and the intestinal bodies of most mice possessed smooth colons with no visible tumors. These results suggested that KGM-CUR/PSM microspheres exhibited a significant synergistic effect on CRC under the dual-response release mechanism of ROS and enzyme.

### 3.11. Histology Analysis

**Figure 11:**
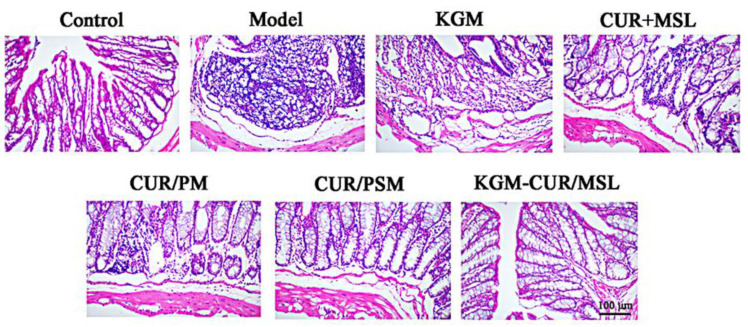
Histopathological analysis of colon tissues from CRC mice (×100).

As shown in Figure 11, the HE staining indicated that colon tissues from the model group showed obvious tumor lesions, structural disorder, inflammatory infiltration, and irregular mass cells compared with samples from the control group. Crypt atrophy, goblet cell injury, and inflammatory infiltration were observed in colonic tissue from the KGM group. However, compared with the model group, pathological phenomena weakened significantly in the colonic tissue from the CUR + MSL and CUR/PM micelle groups, which suggested that CRC could be inhibited by synergistic administration of CUR and MSL to a certain extent. The CUR/PSM micelles group showed a relatively intact structure and mild inflammatory infiltration. Significantly, samples of tissue taken from animals that were being treated with KGM-CUR/PSM microspheres showed that pathological phenomena almost disappeared compared with other groups which demonstrated that the ROS and enzyme dual-response drug delivery system was effective in CRC treatment.

### 3.12. Inflammation Analysis

There is a strong link between inflammation and cancer. During cancer progression, tumor cells secrete numerous cytokines and inflammatory factors into the surrounding areas to form a tumor microenvironment. Therefore, we examined the levels of inflammatory factors in serum and tissues of mice after drug intervention to investigate the anti-inflammatory effects of KGM-CUR/PSM microspheres.

**Figure 12:**
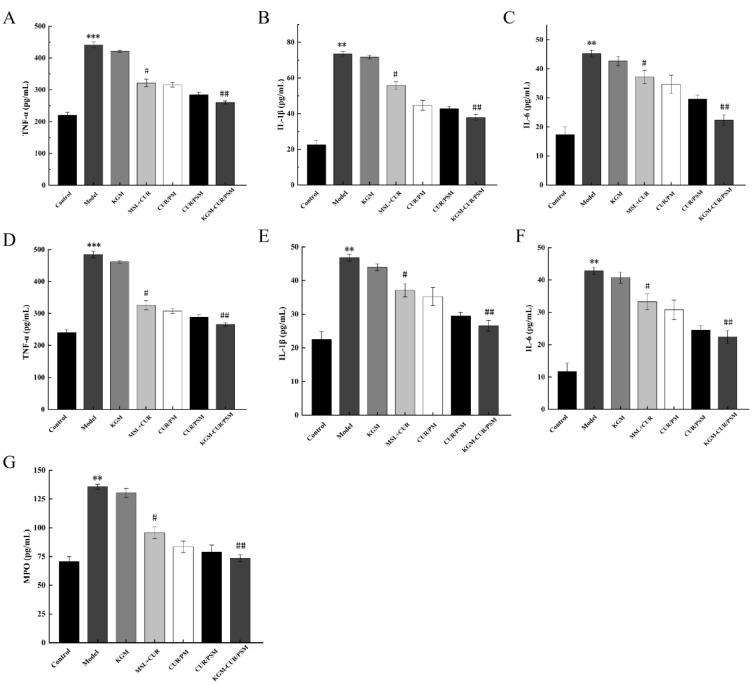
Detection of the expression levels of TNF-α (**A**), IL-1β (**B**), and IL-6 (**C**) in CRC mice (*n* = 5, compared with control, ** *p* < 0.01, *** *p* < 0.001; compared with model, # *p* < 0.05, ## *p* < 0.01). Detection of the expression levels of TNF-α (**D**), IL-1β (**E**), and IL-6 (**F**) in colorectal tissues of CRC mice (*n* = 5, compared with control, ** *p* < 0.01, *** *p* < 0.001; compared with model, # *p* < 0.05, ## *p* < 0.01). MPO expression levels (**G**) in CRC mice, compared with model (*n* = 5, compared with control, ** *p* < 0.01; compared with model, # *p* < 0.05, ## *p* < 0.01).

The expression levels of TNF-α, IL-1β, and IL-6 in mouse serum samples and colorectal tissues were shown in Figure 12A–F. Compared to the control group, the model group exhibited notably elevated levels of TNF-α, IL-1β, and IL-6. However, each administration group showed different degrees of reduction relative to the model group. The anti-inflammatory effect was not obvious in the KGM group. The expression levels of IL-6, IL-1β, and TNF-α in both the CUR group and the MSL group exhibited significant reductions, indicating that both possess favorable anti-inflammatory effects. In the CUR + MSL group, the expression levels of inflammatory factors were reduced, indicating that the CUR and MSL exerted a synergistic anti-inflammatory role, but the therapeutic effect was not as good as that of the micelles group. Owing to the effect of ROS-sensitive bonds, the expression levels of inflammation factors in the CUR/PSM micelles group were lower than those of the CUR/PM micelles group, and the KGM-CUR/PSM microspheres group with a dual-response release mechanism showed the most significant effect on reducing the expression of TNF-α, IL-1β, and IL-6, which could block the inflammatory-cancer transformation through the anti-inflammatory effect, thus enhancing the anti-tumor effect.

Neutrophil infiltration is a hallmark of the inflammatory response, and it is generally proportional to the severity of inflammation. The degree of neutrophil infiltration in colorectal tissue was assessed by measuring MPO activity in mice colorectal tissue, and the results are shown in Figure 12G. Compared with the control group, MPO activity was significantly increased in the model group. However, in the other groups, MPO activity decreased to different degrees after treatment. The KGM-CUR/PSM microspheres group showed the most obvious inhibitory effect on MPO expression, which suggested that KGM-CUR/PSM microspheres could enhance the anti-inflammatory effects of CUR and MSL by inhibiting the expression of inflammatory cells.

### 3.13. Microbiological Analysis

#### 3.13.1. Alterations to the Gut Microbiomes in Different Groups

**Figure 13:**
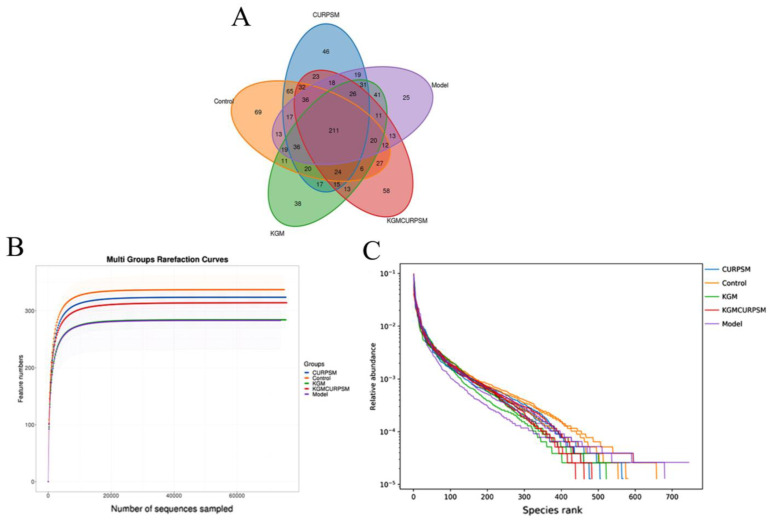

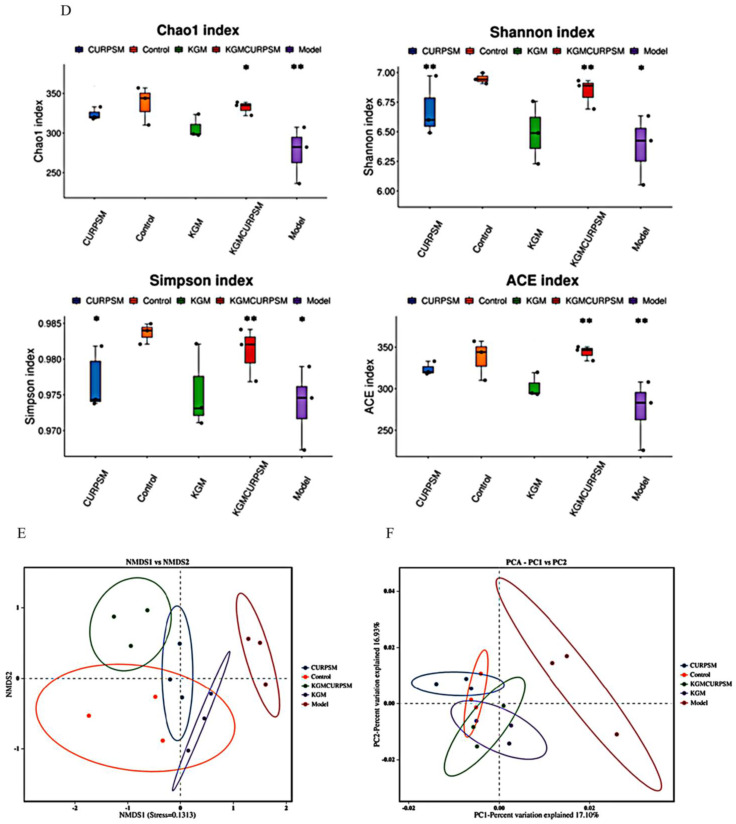
OTU analysis of the gut microbiota (**A**), dilution curves (**B**), and abundance distribution curves (**C**). Alpha diversity index (**D**) Note: compared with control, * *p* < 0.05, ** *p* < 0.01. Non-metric multidimensional scaling analysis (**E**) and principal component analysis (**F**).

To explore the link between tumorigenesis and the gut microbiome, 16S rRNA sequencing was conducted to determine gut microbiota profiles. A total of 211 OTUs were attained among the five groups, including 69 in the control group, 25 in the model group, 38 in the KGM group, 46 in the CUR/PSM micelles group, and 58 in the KGM-CUR/PSM microspheres group (Figure 13A), which indicated that under the dual action of KGM and CUR, the KGM-CUR/PSM microspheres group demonstrated a notable increase in bacteria count and the most obvious improvement in the intestinal microbiota. Moreover, the rarefaction curve was often used to evaluate the saturation of sequencing volumes and sample sizes, which (Figure 13B) showed that the bacterial species tended to flatten as the curve rose, indicating that the sequencing depth could cover all bacterial species. In addition, similar results were obtained for the rank abundance curves (Figure 13C), which suggested that there were no significant differences in species richness and evenness in the experimental groups. To investigate the diversity of intestinal microbiota, we conducted an Alpha analysis. Specifically, the Chao1 and Ace indices were used to reflect species richness, and the Shannon and Simpson indices were employed to indicate species diversity. As shown in Figure 13D, the KGM-CUR/PSM group exhibited the most effective improvement in intestinal microbiota diversity and richness. This suggested that the KGM-CUR/PSM microspheres, through the synergistic action of CUR, MSL, and KGM, significantly improved the intestinal environment, thereby enhancing anti-inflammatory effects and resistance to colorectal cancer. Non-metric multidimensional scaling analysis (Figure 13E) revealed that the model group deviated significantly from the control group, indicating that the microbiota composition of CRC mice was greatly different from that of the control group. The principal component analysis (Figure 13F) showed that the model group was significantly deviated from the other groups, while the administered group was close to the normal group. KGM-CUR/PSM microspheres were the most obvious, demonstrating that KGM-CUR/PSM microspheres had the effect of restoring and improving the intestinal microbiota structure, and showed a significant therapeutic effect on CRC.

#### 3.13.2. Analysis of the Structure of Intestinal Bacteria

**Figure 14:**
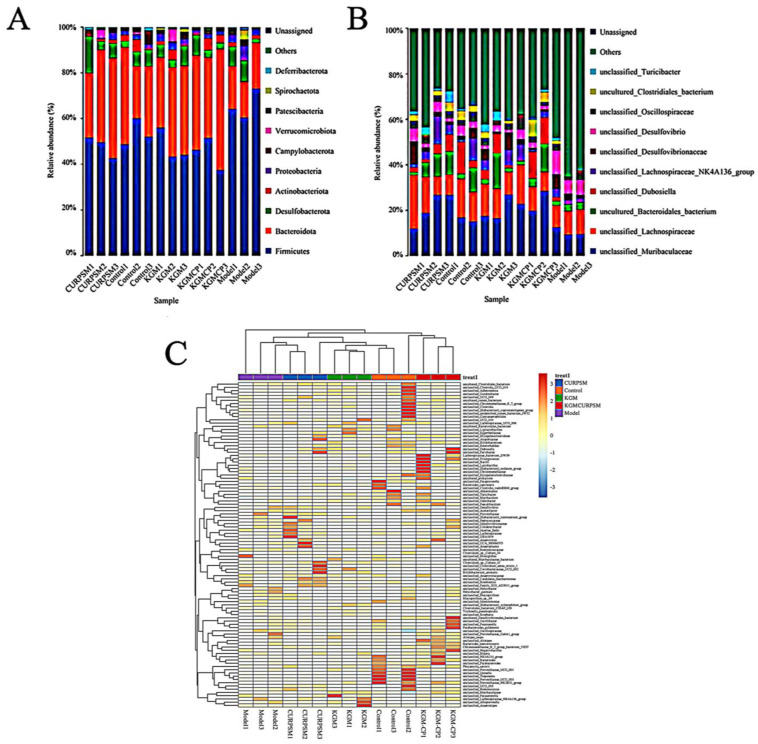
Relative abundance at the phylum level, showing dominance of Firmicutes and Bacteroidota in all groups, with increased Proteobacteria (a pathogenic phylum) in the model group (**A**), genus-level distribution, highlighting restoration of beneficial genera (e.g., Turicibacter, Lachnospiraceae) and suppression of harmful bacteria (e.g., Desulfovibrio) by KGM-CUR/PSM treatment (**B**), and genus-level species composition heat map of species clustering (**C**).

It was well-documented that alterations in the gut microbiota may play a role in the initiation and progression of CRC. A major feature of intestinal dysbiosis is the change in the composition of the intestinal microbiota, which is manifested by the decrease in beneficial bacteria and the increase in harmful bacteria. The structures of the gut microbial communities at both the phylum and genus levels were examined across all groups. The phylum level of mice (Figure 14A) consisted mainly of Firmicutes, Bacteroidota, Verrucomicrobiota, Proteobacteria, etc. The genus level of mice (Figure 14B) consisted mainly of Turicibacter, Lachnospiraceae, Dubosiella, etc. Then, the differential component proportions of different strains of bacteria in each group were displayed based on the heatmaps (Figure 14C). At the phylum level, we observed that the Firmicutes and Bacteroidetes were the dominant bacteria, which were significantly enriched in each group, with the proportion reaching more than 80%. In addition, proteobacteria, the intestinal commensal that accelerated the deterioration of CRC, were significantly enriched in the model group. At the genus level, we observed that the comparative abundance of beneficial bacteria in the model group decreased compared with the control group such as Turicibacter, Lachnospiraceae, Dubosiella, and Clostridiales, while the relative abundance of harmful bacteria or inflammation-associated bacteria increased such as Desulfovibrio and Desulfovibrionaceae. Turicibacter is a crucial member of the mammalian gut microbiota and is involved in fermentative metabolism, which is associated with changes in dietary fat and body weight; Lachnospiraceae are able to hydrolyze starches and other sugars to produce butyrate and other short-chain fatty acids, which could prevent colorectal cancer by producing butyric acid; Clostridiales could suppress the proliferation and replication of harmful and spoilage bacteria in the intestinal tract, correct intestinal disorders, and at the same time produce extracellular soluble polysaccharides with anti-tumor effects; Dubosiella regulates the body’s metabolism, improves intestinal immunity, reduces the production of intestinal inflammatory factors and inhibits the proliferation of related cells. However, Desulfovibrio could destroy intestinal epithelial cells and induce inflammation, leading to gastrointestinal disorders. The results indicated that all treatment groups suppressed harmful bacteria and increased beneficial bacteria to varying degrees, but the KGM-CUR/PSM microspheres group showed the best therapeutic effect. KGM hydrolysis product contains prebiotics beneficial to intestinal microbiota, which could regulate intestinal microbiota and improve the intestinal microenvironment. Meanwhile, MSL could reduce intestinal inflammation and maintain the balance of intestinal microbiota. Under these combined effects, the KGM-CUR/PSM microspheres group significantly adjusted the relative abundance of the above genera to varying degrees, and the results were close to those of the control group, which inhibited the harmful bacteria, up-regulated the beneficial bacteria, and showed the best therapeutic effect. The microbiota composition in the model group was partially restored after treatment, indicating that KGM-CUR/PSM microspheres could regulate the composition of microbiota to improve the intestinal environment.

## 4. Discussion

In recent years, the design of stimulus-responsive drug delivery systems leveraging the characteristics of the tumor microenvironment (e.g., pH, enzyme expression, oxidative stress levels) has emerged as a research hotspot in cancer therapy. For instance, pH-sensitive polymeric micelles, reduction-responsive prodrugs, and enzyme-triggered nanoparticles can achieve precise drug release at tumor sites, thereby enhancing therapeutic efficacy while minimizing systemic toxicity. The KGM-CUR/PSM microspheres developed in this study are similar to these strategies but exhibit unique ROS/enzyme dual-responsive properties. This design not only improves the specificity of drug release in CRC tissues but also achieves multifaceted therapeutic effects by combining the anti-tumor activity of CUR with the anti-inflammatory properties of MSL.

Notably, although numerous nano-drug delivery systems have been developed for CRC treatment, most focus solely on targeted drug delivery and anti-tumor effects, overlooking the critical roles of chronic inflammation and gut microbiota dysbiosis in the CRC microenvironment. This study provides a more comprehensive CRC treatment strategy by integrating the anti-inflammatory drug MSL with KGM, a gut microbiota modulator.

The initiation and progression of CRC are closely associated with chronic inflammation, where inflammatory cytokines such as TNF-α, IL-1β, and IL-6 play pivotal roles in tumorigenesis and metastasis. The results of this study demonstrate that, compared to the model group, KGM-CUR/PSM microspheres significantly reduced the expression levels of TNF-α, IL-1β, and IL-6 in serum and colon tissues. These findings not only validate the efficacy of MSL as an anti-inflammatory agent but also suggest that the combination of CUR and MSL exerts synergistic anti-inflammatory effects, thereby more effectively disrupting the “inflammation-cancer” transformation process.

Gut microbiota dysbiosis is another key factor in CRC development. A decrease in beneficial bacteria and an increase in harmful bacteria can compromise intestinal barrier function, exacerbating inflammatory responses and tumorigenesis. In this study, 16S rRNA sequencing was employed to analyze the gut microbial composition of mice across different treatment groups. The results revealed that KGM-CUR/PSM microspheres significantly enhanced gut microbial diversity and richness, restored the relative abundance of beneficial bacteria (e.g., Turicibacter, Lachnospiraceae, and Dubosiella), and suppressed the proliferation of harmful bacteria (e.g., Desulfovibrio). These changes were closely correlated with improvements in the intestinal microenvironment and enhanced anti-tumor efficacy. As a natural polysaccharide, KGM not only exhibits enzyme-degradable properties but also acts as a prebiotic to promote the growth of beneficial bacteria. By incorporating KGM into the drug delivery system, this study achieved targeted drug release while modulating the gut microbiota to improve the intestinal microenvironment, thereby amplifying the anti-tumor effects.

In summary, this study successfully developed a ROS/enzyme dual-responsive drug delivery system, KGM-CUR/PSM microspheres, for the precise treatment of CRC. Through in vitro and in vivo experiments, the system’s multifaceted effects—including anti-tumor, anti-inflammatory, and gut microbiota-modulating activities—were comprehensively validated. The system demonstrates significant advantages in both drug release precision and therapeutic outcomes, offering novel insights and methodologies for the clinical management of CRC.

## 5. Conclusions

In conclusion, this study has successfully developed a ROS/enzyme dual-responsive drug delivery system, KGM-CUR/PSM microspheres, designed for targeted CRC therapy. By leveraging the unique tumor microenvironment characteristics of CRC, including elevated ROS levels and specific enzyme expression, we constructed a stimuli-responsive prodrug system that achieved precise drug release at the tumor site. The system combined the anti-tumor effects of CUR with the anti-inflammatory properties of MSL, encapsulated within KGM microspheres, to enhance therapeutic efficacy through synergistic chemotherapy, anti-inflammation, and gut microbiota modulation.

The outcomes of in vitro release experiments demonstrated that the KGM-CUR/PSM microspheres could be accurately localized and released in the colorectal, which could efficiently increase the enrichment of CUR and MSL in colorectal tumor cells. In vivo experiments showed that KGM-CUR/PSM microspheres effectively inhibited the growth of colorectal tumors, down-regulated the expression of TNF-α, IL-1β, and IL-6 in serum and tissues, inhibited the production of MPO enzyme and pro-inflammatory bacteria, reduced the damage to the intestine, adjusted the ratio among microbiota, and restored the richness and diversity of intestinal microbiota in vivo. Under the synergistic effect of CUR, MSL, and KGM, the growth, proliferation, and migration of SW480 cells were inhibited. This study established a multi-mechanism synergistic CRC treatment system, which integrated apoptosis induction, anti-inflammation, and microbiota modulation to form a trinity therapeutic strategy of “tumor suppression-anti-inflammation- microenvironment remodeling”, significantly enhancing the therapeutic efficacy and safety for CRC while providing novel insights for CRC pharmacotherapy.

## Figures and Tables

**Table 1:** Summary of Compounds Used in the Study.

Compound	Characteristics
Curcumin (CUR)	Natural lipophilic polyphenol from Curcuma longa; molecular weight: 368.38; poor water solubility; low bioavailability; anti-CRC; anti-inflammatory; antioxidant; gut microbiota modulator.
Mesalazine (MSL)	5-aminosalicylic acid, a non-steroidal anti-inflammatory drug; molecular weight: 153.14; inhibits cyclooxygenase (COX), reduces prostaglandin production; suppresses CRC via Cyclin D1; PPAR-γ/AMPK activation; Wnt/β-catenin inhibition.
Polyethylene glycol monomethyl ether (mPEG)	Hydrophilic polymer (MWCO = 1900 Da); excellent biocompatibility; forms hydrophilic shell of micelles.
Konjac glucomannan (KGM)	Natural polysaccharide carrier; colon-specific degradation by β-mannanase; promotes intestinal peristalsis; alleviates inflammation and modulates microbiota.
2,2′-Thiodiacetic acid (TDA)	Precursor for ROS-sensitive linker; contains thioether bond (-S-).
Azoxymethane (AOM)	Carcinogen used to induce CRC in mice.
Dextran sodium sulfate (DSS)	Colitis-inducing agent; used with AOM to establish CRC mouse model.
4-dimethylaminopyridine (DMAP)	Catalyst; molecular weight: 122.17;
N,N′-dicyclohexylcarbodiimide (DCC)	Catalyst; molecular weight: 206.33;
N-hydroxysuccinimide (NHS)	Catalyst; molecular weight: 115.09;
Glutaraldehyde	Cross-linking agent.

**Table 2:** Inhibition rates of CUR and MSL alone or in combination at varying molar ratios.

Conc.(μM)	CUR Alone	MSL Alone	0.5:1 (CUR:MSL)	1:1 (CUR:MSL)	1.5:1 (CUR:MSL)	2:1 (CUR:MSL)	2.5:1 (CUR:MSL)
10	22.0%	0.0082%	22.8%	25.7%	31.0%	28.0%	22.6%
20	30.0%	0.0143%	31.7%	40.0%	45.3%	42.0%	33.8%
30	37.0%	0.02105%	38.3%	47.7%	51.7%	49.5%	39.8%
40	44.0%	0.02559%	45.3%	51.0%	58.6%	53.1%	45.6%
50	49.0%	0.029%	49.7%	56.2%	62.0%	58.3%	51.3%

Note: Conc. (μM): Total concentration of CUR and MSL in combined treatments. For single-drug groups (CUR alone, MSL alone), concentration refers to the individual drug.

## Data Availability

The data that support the findings of this study are available from the corresponding author upon reasonable request.
